# Cell-Type Specific Penetrating Peptides: Therapeutic Promises and Challenges

**DOI:** 10.3390/molecules200713055

**Published:** 2015-07-20

**Authors:** Maliha Zahid, Paul D. Robbins

**Affiliations:** 1 Research Instructor, Department of Developmental Biology, University of Pittsburgh, Pittsburgh, PA 15201, USA; 2 Excela Health Cardiology, Greensburg, PA 15601, USA; 3 Department of Metabolism and Aging, Scripps Florida, Jupiter, FL 33458, USA; E-Mail: PRobbins@scripps.edu

**Keywords:** cell-penetrating peptides, protein transduction domains, phage display, biopanning

## Abstract

Cell penetrating peptides (CPP), also known as protein transduction domains (PTD), are small peptides able to carry peptides, proteins, nucleic acid, and nanoparticles, including viral particles, across the cellular membranes into cells, resulting in internalization of the intact cargo. In general, CPPs can be broadly classified into tissue-specific and non-tissue specific peptides, with the latter further sub-divided into three types: (1) cationic peptides of 6–12 amino acids in length comprised predominantly of arginine, lysine and/or ornithine residues; (2) hydrophobic peptides such as leader sequences of secreted growth factors or cytokines; and (3) amphipathic peptides obtained by linking hydrophobic peptides to nuclear localizing signals. Tissue-specific peptides are usually identified by screening of large peptide phage display libraries. These transduction peptides have the potential for a myriad of diagnostic as well as therapeutic applications, ranging from delivery of fluorescent or radioactive compounds for imaging, to delivery of peptides and proteins of therapeutic potential, and improving uptake of DNA, RNA, siRNA and even viral particles. Here we review the potential applications as well as hurdles to the tremendous potential of these CPPs, in particular the cell-type specific peptides.

## 1. Introduction

The plasma membrane of cells is an effective semi-permeable barrier that is essential to cell integrity and survival and at the same time presents a barrier to intracellular delivery of potentially diagnostic or therapeutic cargo. Hence the ability of certain proteins to cross cell membrane barriers, a process termed protein transduction, described over 25 years ago has great research and clinical utility. This finding was first reported in 1988 by two separate groups who demonstrated the ability of Trans-Activator of Transcription (Tat) protein of the Human Immunodeficiency Virus (HIV) to enter cultured cells and promote viral gene expression [1,2]. Additionally, it was shown that Antennapedia homeodomain (Antp), a homeobox transcription factor of *Drosophila* *melanogaster*, could enter nerve cells and regulate neural morphogenesis [3]. Mapping of the domains able to confer this transduction ability of these proteins led to the identification of first two cell penetrating peptides (CPP): Tat peptide corresponding to the 11 amino acid basic domain of HIV-1 Tat protein [4] and penetratin, corresponding to the 16 amino acid third helix of the Antennapedia domain [5]. Subsequently, the ability of full-length TAT chemically cross-linked to multiple different proteins, ß-galactosidase, horseradish peroxidase, RNase A and domain III of Pseudomonas exotoxin A (PE), to transduce multiple different cell types was demonstrated [6]. Moreover, the ability of this 11-amino acid Tat transduction peptide to carry cargoes as large as 120 kDa across cell membranes as well as cross the blood-brain barrier was demonstrated [7], highlighting the therapeutic potential of these unique peptides. Since the initial characterization of these two transduction domains, the number of peptides identified as having cell penetrating properties have expanded exponentially as has the number of publications using CPPs. Therefore, this review will give a broad overview of classification, mechanism of transduction, potential diagnostic and therapeutic applications of CPPs.

## 2. Classification

Although CPPs have a great sequence variety with little homology or overlap, they can be broadly divided into tissue-specific and non-tissue specific peptides. Non-tissue specific can be further classified into three classes: cationic, hydrophobic and amphipathic CPPs. The very first CPPs identified, Tat and penetratin, are both cationic peptides. In addition to these naturally occurring peptide sequences, additional cationic peptides include homo-polymers of Arginine [8], Lysine [9], and the herpes-simplex-virus-1 DNA binding protein VP22 [10] (Table 1). Studies on Arginine based homo-polymers (from R3 to R12 length), have shown that the minimal sequence necessary for cellular uptake was six arginine, and that increasing the number of Arginine residues, increased transduction efficiency [11]. Poly-Lysine, in comparison, demonstrated reduced uptake [11] in this particular study, though we have reported similar cellular uptake with both 8-mer homo-polymers of lysine or arginine [12]. However, Arginine and Lysine homo-polymers larger than 12 amino acids show reduced transduction efficiency.

Amphipathic CPPS are chimeric peptides, several of which were obtained by covalent attachment of a hydrophobic domain to a nuclear localizing signal (NLS). Two such peptides, MPG (GLAFLGFLGAAGSTMGAWSQPKKKRKV) and Pep-1 (KETWWETWWTEWSQPKKRVK) are both based on the SV40 nuclear localizing signal PKKRKV. Other primary amphipathic CPPs are fully derived from natural proteins, such as pVEC [13], ARF (1-22) [14], and BPrPr (1-28) [15].

**Table 1:** Classification of cell penetrating peptides.

**Types of CPPs-Non-Tissue Specific**	**Sequence**	**Source**
Cationic		
TAT [4]	GRKKRRQRRRPPQ	HIV Tat Protein
Ant [5]	RQIKIWFQNRRMKWKK	Antennapedia homeodomain
8-Arginine [8]	RRRRRRRR	n/a
8-Lysine [9]	KKKKKKKK	n/a
PTD-5 [12]	RRQRRTSKLMKR	Phage display
Hydrophobic		
Transportan [16]	GWTLNSAGYLLGKINLKALAALAKKIL	Galanin and mastoparan
MAP [17]	KLALKLALKALKAALKLA	
TP10 [18]	AGYLLGKINLKALAALAKKIL	Galanin and mastoparan
Amphipathic		
Azurin p18 [19]	LSTAADMQGVVTDGMASG	Azurin
Azurin p28 [20]	LSTAADMQGVVTDGMASGLDKDYLKPDD	Azurin
hCT18-32 [21]	KFHTFPQTAIGVGAP	Calcitonin
**Types of CPPs-Tissue Specific**	**Sequence**	**Source**
CTP [22]	APWHLSSQYSRT	Phage display
K5-FGF [23]	AAVALLPAVLLALLP	Phage display
HAP-1 [24]	SFHQFARATLAS	Phage display
293P-1[25]	SNNNVRPIHIWP	Phage display

Hydrophobic CPPs are derived from signal peptide sequences or identified through phage display, plasmid display, microorganism surface display and ribosome display of large peptide libraries. The hydrophobic transduction peptides identified include leader sequences for KGF and FGF. Related types of CPPs include amphipathic, chimeric peptides, several of which were obtained by covalent attachment of a hydrophobic domain to a nuclear localizing signal (NLS) rich in positively charged amino acids. Two such peptides, MPG (GLAFLGFLGAAGSTMGAWSQPKKKRKV) and Pep-1 (KETWWETWWTEWSQPKKRVK) are both based on the SV40 nuclear localizing signal PKKRKV. Other primary amphipathic CPPs are fully derived from natural proteins, such as pVEC [13], ARF (1-22) [14], and BPrPr (1-28) [15].

The ability of cationic or hydrophobic CPPs to transduce a wide variety of tissue types *in vivo* limits their utility because of lack of cell selectivity, leading to a higher chance of off-target, non-specific effects, thereby increasing the likelihood of adverse side effects. To circumvent the problem of non-specificity, there are two distinct approaches: identify peptides with tissue specific transduction abilities or restrict the ability of the non-tissue specific CPP bearing cargo in a pro-drug fashion that is activated only in certain cell types. Identifying CPPs selective for specific cell-types is particularly attractive as it has the potential for delivering therapeutic cargoes in higher concentrations to the tissue of interest, for example solid tumors, while limiting non-specific uptake, toxicity as well as total dose of certain drugs. The dose issue becomes particularly important in regards to scaling up from animal to human studies and thus dictating the total cost of a potential therapeutic.

## 3. Tissue Specific Transduction Peptides

To identify tissue specific transduction peptides, phage display, using combinatorial libraries of various lengths and different phages, has been utilized successfully. Phage display involves exposing the target cell type or tissue of interest to a large, randomized combinatorial library of phage in which one of the envelope proteins has been modified to carry peptides of different lengths [26]. The phage that bind to or are internalized into specific cells can be isolated, expanded and used in another round of screening. Usually 4–5 rounds of screening results in the identification of small number of peptides able to facilitate binding and/or internalization of intact phage into the target cells. The strength of this method lies in that *a priori* knowledge of the target is not necessary. For example, using *in vivo* phage display Pasqualini and colleagues were able to identify NRG and RGD motifs able to target phage to tumor vasculature in nude mice bearing breast carcinoma, human Kaposi’s sarcoma and mouse melanoma xenografts [27]. In addition, the chemotherapeutic agent, doxorubicin, could be coupled to peptides containing repeats of the above motifs, resulting in therapeutic benefit in the form of decreased tumor size and increased survival when compared to doxorubicin alone [27]. Subsequently, multiple studies have reported use of phage display to identify peptides able to target vascular endothelium [28], synovial tissue [24], dendritic cells [29], pancreatic islet cells [30] and cardiac myocytes [22].

We employed a combinatorial approach of *in vitro* one cycle of screening an M13 phage display library against H9C2 cells, a rat cardiomyocyte cell line, and used the recovered, enriched phage in subsequent cycles of *in vivo* phage display [22]. After a total of five cycles, a predominant peptide emerged, which we termed cardiac targeting peptide (CTP). CTP was able to transduce mouse heart tissue rapidly and efficiently after an intra-venous injection with peak transduction seen at 30 min, with very little uptake by skeletal muscle tissue, liver, spleen or lungs [22]. Subsequently, CTP was used to direct photodynamic therapy to cardiac myocytes while sparing other cell types in culture [31]. In another application, CTP was used to deliver Cre-recombinase protein to heart tissue *in vivo* in mice [32].

Other approaches to facilitate targeted drug delivery using CPPs consist of using a non-targeting CPP to deliver cargo with targeted activity. For example, selective killing of HIV infected cells was demonstrated by linking TAT to proCaspase-3 where the endogenous cleavage site was substituted for an HIV proteolytic cleavage site [33]. Administration of this peptide resulted in transduction of various different cell types, but apoptosis only of the HIV infected cells. Building on this approach, Harada and colleagues took advantage of the hypoxic environment of tumors resulting in induction of hypoxia-inducible transcription factor Hif-1α [34]. This factor contains an oxygen-dependent degradation domain, rendering the protein unstable in aerobic environment. Engineering this domain into TAT with proCaspase-3 induced apoptosis in cells growing under hypoxic conditions and intraperitoneal injection led to suppression of tumor growth [34].

## 4. Mechanism of Transduction

The mechanism(s) used by CPPs to traverse cell membranes remains an area of active study. Although the exact mechanism(s) remains poorly defined, nevertheless the two proposed major cellular uptake mechanisms of CPP include a non-endocytic, energy independent as well as an endocytic, energy dependent pathway with evidence existing for both. It is not known whether the cellular entry of CPPs entails use of specific cellular receptors although it is known that cationic CPPs interact with glycosaminoglycans (see below) on the cell surface. It is likely that the mechanism of transduction varies from one CPP to another, and even for each CPP, depending on the specific cargo it is loaded with or fused to as well as change in physiological parameters such as concentrations, local milieu and pH. In addition, recent data suggests that CPPs might be using more than one pathway and one may be preferentially chosen over another depending on the concentration of the peptide at the cell membrane [35,36,37].

The broad range of cell types that are readily transducible with these CPPs suggests an involvement of ubiquitously shared cellular structures such as surface binding to plasma membrane phospholipids. For example, binding of cationic CPPs to heparan sulfate proteoglycans is important for subsequent transduction [9,38,39]. Following this binding, cationic CPPs with small cargoes likely enter cells quickly via direct translocation in addition to the endocytic pathway, whereas uptake of larger cargoes attached to these peptides might be via micropinocytosis in an energy dependent manner and at a slower rate [40]. Transduction can occur at 4 °C and after depletion of the ATP pool [9], although at greatly reduced levels, suggesting that it is not completely an energy-dependent process. Results also suggest that increasing the hydrophobic characteristics of the CPP increases its efficiency as a transporter, as seen in the case of TAT [41]. It is possible that for any CPP multiple transduction mechanism may be involved or that the mechanism varies between cells types as well as between CPPs, and is most likely to change depending on the cargo of interest being delivered. 

## 5. Therapeutic Applications of CPPs

Given the large number of CPPs identified to date, as well as the wide number of cells and tissues they are able to transduce, a comprehensive summary of all their applications in one review would be impossible. Here we describe a selected sample of some of the reported applications. 

### 5.1. Targeting NF-κB

Nuclear factor kappa B (NF-κB) is a transcription factor known to regulate expression of genes involved in the inflammatory response. Among the many stimulators of NF-κB activation are pro-inflammatory cytokines such as tumor necrosis factor (TNF) and interleukin-1 (IL-1). NF-κB is also activated by genotoxic and oxidative stress as well as by viral infection and mechanical stress. NF-κB activation is rapid, but transient in response to the inducing stimuli leading to a time-limited course of potentially damaging, inflammatory or apoptotic gene activation. However, dysregulated and constitutively active NF-κB activation has been reported in chronic inflammatory disorders ranging from rheumatoid arthritis [42], atherosclerosis [43], Parkinson’s disease [44], inflammatory bowel disorders [45] and aging [46], as well as enhancing tumor tissue survival in cancer [47]. Activation of NF-κB in response to stimulation requires release of inhibitory effects of IκB, by a kinase complex comprised of a regulatory subunit, NEMO (NF-κB essential modulator) and two catalytic subunits, IKKα and IKKß. Biochemical analysis revealed that a peptide spanning the domain NEMO uses to bind to IKKß, TALDWSWLQTE, was able to block the interaction of NEMO with this complex, inhibiting the normal activation of NF-κB in response to appropriate inflammatory stimuli [48]. Interestingly, a fusion peptide containing the Antp CPP followed by this 11 amino acid Nemo-binding domain (NBD) was able to penetrate cells and inhibit NF-κB activation in response to appropriate stimuli [48]. These findings have led to a multitude of studies being conducted to treat different disorders with a common inflammatory basis with the NBD peptide in combination with one of many different CPPs being used to deliver this cargo [49]. 

Duchenne muscular dystrophy (DMD) is the most common, X-linked, recessive disorder, resulting from genetic deficiency of dystrophin in myocytes. In these patients, as well as *mdx* mice, a mouse model of DMD, there is increase in NF-κB activation, muscle necrosis and chronic inflammation in addition to reduced muscle regeneration. Chronic treatment of young mdx mice by intraperitoneal injection of an Antp-NBD fusion peptide resulted in decreased NF-κB activation, decreased necrosis and increased muscle regeneration in hind limbs and diaphragm muscles [50,51,52]. In another report utilizing *mdx* mice, treatment with 40–200 µg/mouse of NBD peptide linked to Ant, 3×/week, intraperitoneally, rescued 78% of the contractile deficit between *mdx* and wild-type diaphragm [52]. Delfin and colleagues demonstrated that delivery of the Ant-NBD peptide intraperitoneally to a double knock-out mouse model of DMD lacking both dystrophin and its homologue utrophin improved cardiac function more than double that of untreated mice [53]. Treatment of the golden retriever dog model (GRMD) of DMD with Antp-NBD resulted in an improvement in pelvic limb muscle force and in histopathologic lesions [54]. The NBD-treated GRMD dogs also had normalized postural changes with lower tissue injury. These results suggest that CPP-NBD fusion peptide could be used clinically to treat DMD. However, the dog studies also demonstrated that chronic Antp-NBD treatment resulted in infusion reactions and an immune response in both GRMD and control dogs. Whether a similar immune response would be observed with other CPPs is unclear.

Inflammatory bowel disease (IBD), comprised of Crohn’s disease and Ulcerative colitis, is a chronic inflammatory bowel disease with sustained up-regulation of inflammatory mediators such as TNF-α, IL-6, and IL-1, which themselves are regulated by the activation of NF-κB. Therefore inhibiting a transcription factor like NF-κB would reduce expression of these key inflammatory factors, which should result in reduction or amelioration of the disease activity. Indeed, intraperitoneal injection of NBD-peptide fused to an 8-Lysine CPP was able to inhibit NF-κB activation in response to lipopolysaccharide in macrophages and reduce NF-κB activation and disease pathology in an IL-10^−/−^ mouse model of IBD [55]. This study demonstrates the ability to inhibit NF-κB activation and treat IBD through systemic delivery of the NBD peptide using CPPs.

More recently, investigators have utilized intra-nasal delivery of TAT-NBD peptide to rat models of infection-associated brain hypoxic-ischemic injury [56]. Not only did the fusion peptide transduce the olfactory bulb rapidly (10–30 min), its uptake peaked in the cerebral cortex at 60 min, markedly reducing the NF-κB signaling, microglial activation and brain damage triggered by hypoxic-ischemia insult. In addition, these effects were observed at only 7% of the full intravenous dose. This study demonstrates how a non-specific, ubiquitously transducing cationic CPP like TAT can still be used in a tissue specific manner by utilizing different, more localized routes of administration. 

### 5.2. Therapeutic Implications for Cancer Treatment

CPPs have been used extensively to deliver chemotherapeutic agents or biologics to target tumors [57]. In particular, the anti-tumor activity of therapeutic agents has been enhanced by taking advantage of processes unique to the tumor cells. For example, phospholipase C-gamma 1 activity in tumor cells was reduced by conjugating an inhibitor consisting of a PLC-gamma1 SH2 domain to the TAT peptide [58]. This conjugate was able to block the metastasis and spread of EGFR/*c-erb*-2-positive breast cancer cells. The tumor suppressor protein, p53, was introduced into oral cancer cell lines by conjugating it to a poly-arginine transduction peptide [59]. The magnitude of the anti-tumor effect observed was equivalent to that seen with adenoviral-mediated p53 gene delivery. A further refinement of this approach involved linking the polyarginine-p53 fusion protein to the NH2-terminal domain of influenza virus hemagglutinin-2 subunit [60]. This modification allowed a pH-dependent lysis of endosomal membranes, with efficient translocation into nucleus of glioma cells and inhibition of cancer cell growth at low doses. Similarly, the D-isomer of the p53 C-terminus, presumably able to interact with and modulate the conformation of mutant p53, connected to a retro-inverso version of the NH2-terminal 20-amino acid peptide of the influenza virus hemagglutinin-2 protein with a poly-arginine CPP, was able to inhibit growth of bladder cancer after a single application [61]. In addition, small pro-apoptotic peptide consisting of the BH3 biological effector domain of the pro-apoptotic Bcl-2 family member, Bim, have been successfully delivered to T-cell lymphoma cell line, pancreatic cancer and melanoma cell line using TAT as a carrier peptide, resulting in significant reduction in tumor growth in murine models [62]. In another approach, TAT was fused to a pro-apoptotic peptide, KLAKLAK, conjugated with a caspase-3 site [63]. This novel peptide was able to induce tumor cell apoptosis *in vitro* and *in vivo*, inhibit angiogenesis in endothelial cells, and led to almost complete inhibition of melanoma tumor cell growth.

An alternative approach to improve CPP-based targeted cancer therapy has been to develop tumor-homing or tumor selective peptides. The feasibility of this approach was demonstrated through the use of phage display to identify RGD containing peptides able to home to specific integrins expressed preferentially in the tumor vasculature [27]. Furthermore, the RGD motifs could be conjugated to chemotherapeutic agent doxorubicin, enhancing its efficacy against human breast cancer xenografts in nude mice with concomitant reduction in its toxicity and increased survival. In another approach, the fusion of the amino terminal hydrophobic domain, MIIYRDLISH, of a translationally controlled tumor protein fused to the pro-apoptotic peptide (KLAKLAK)_2_ resulted in efficient transduction of tumor cells, increased apoptosis and decreased cell viability [64]. The tumor inhibitory effect of this fusion peptide was even better than TAT-KLAK in a xenograft of lung carcinoma in Balb/c nude mice [64].

### 5.3. Viral and Non-Viral Gene Delivery

Successful viral infection of a target cell requires efficient binding of the viral particles to the plasma membrane before entry into the cell. Enhancing the efficiency of viral transduction by conjugating the viral membrane binding proteins with a transduction domain is another application for CPPs [65]. Adenoviral vector transfection is limited to cells bearing the primary adenovirus receptor or the Coxsackie virus and adenovirus receptor (CAR) with low transfection in CAR-negative cells. Adenoviral infection efficiency can be increased by pre-incubating the virus with Antp CPP, both *in vitro* and *in vivo* [66]. Other investigators have chemically conjugated various CPPs, such as TAT or octa-arginine to recombinant adenovirus, resulting in 1–2 log orders higher expression than the unmodified adenovirus in CAR-negative cells [67]. A novel CPP derived from herring protamine conjugated to adenovirus enhanced the efficiency of transfection of a rat glioma cell line to a greater extent than other known CPPs such as TAT or Antp [68]. Moreover, this novel peptide-recombinant adenovirus conjugate was able to enhance viral transfection of mesenchymal stem cells and dendritic cells, which are relatively resistant to adenoviral infection.

In addition to improving viral infection, CPPs have been used to enhance non-viral mediated gene delivery [69,70]. For example, fusion proteins containing the TAT peptide followed by DNA-binding motifs were complexed with reporter gene DNA alone or DNA and cationic liposomes [54]. Analysis by confocal microscopy and immuno-staining demonstrated efficient uptake and nuclear localization of the DNA in multiple different cell lines *in vitro*. Addition of cationic liposomes enhanced efficiency further by 5–7 folds. In addition to DNA, CPPs have been utilized to deliver small interfering RNA (siRNA) to cell lines. Since the discovery of intracellular administration of short, synthetic, double-stranded RNA duplexes to lead to down-regulation of specific mRNA expression [71], significant effort has been placed on developing efficient means of intracellular delivery. As these siRNAs have a strong anionic charge, a phosphodiester backbone and an approximate size of ~14,000 Da, they are unable to penetrate the phospholipid bilayer of cells. The initial studies utilized a covalent conjugation of various CPPs with siRNA *in vitro* in HeLA cells, and showed biological effects in terms of down-regulation of p38 kinase mRNA levels, but at a 1000-fold to a 100,000-fold higher concentration than lipofectamine alone [72,73]. After these initial disappointing results that used covalent conjugation of siRNA with CPPs, Ezzat and colleagues demonstrated the ability of a cationic CPP to bind to siRNA in a non-covalent fashion and functionally deliver the siRNA to cells at low doses [74,75]. Also, a synthetic polypeptide able to assume helical structure independent of pH was developed, allowing the synthetic peptide to assume this structure at physiological pH to facilitate endosomal escape and enhance siRNA delivery to mammalian cells [76]. Whether these approaches of bypassing a viral vector and conjugating nucleic material directly to CPPs will result in a higher efficiency of gene delivery *in vivo* remains to be seen. 

## 6. Imaging Applications

The use of CPPs has also been applied to imaging approaches *in vivo*. Here, technology similar to that developed for cancer therapeutics has been utilized. For example, activatable CPPs consisting of the peptide with transduction ability fluorescently labeled and coupled via a cleavable linker to a neutralizing peptide have been utilized. The linker contains a cleavable site recognized by proteases that are express by tumor tissue. Upon exposure to tumor tissue and its associated proteases, the neutralizing peptide is cleaved off, providing a locally high concentration of the CPP and resulting in enhanced uptake by tumor tissue [77]. Utility of this technique has been shown to label tumor tissue, better delineate the margins between tumor and normal tissue and result in improved precision of tumor resection [77]. Alternatively, parallel *in vivo* and *in vitro* phage display can be used to identify protease-dependent tumor targeting peptides. This approach demonstrated that the most efficiently cleaved peptide contained the substrate sequence RLQLKL, able to label tumor tissue as well as metastases from several cancer models with up to a five-fold contrast enhancement [78].

Quantum dots (QD) are brightly fluorescent, photostable, semiconductor nanocrystals, 1–6 nm in diameter that are being use more extensively for biological imaging. Advantages of QDs over traditional dyes consist of high quantum yield, narrow emission peak, excellent resistance to photo-bleaching and broad, size-dependent photoluminescence. Nevertheless, they are limited by their poor ability to translocate across the plasma membrane. CPPs, most commonly TAT, has been utilized to overcome this limitation. Early dynamic confocal imaging studies showed that TAT-QD conjugates were internalized by macropinocytosis triggered by the binding of the conjugate to negatively charged cell membranes. The internalized TAT-QD was tethered to the inner vesicle surface and trapped in cytoplasmic organelles [79]. In addition, cells can be labeled in culture using TAT-QD conjugates for analysis of cell fate following injection. In particular, a TAT-QD conjugate was used to successfully label stem cells and track the fate of these cells injected intravenously into NOD/SCID null mice [80]. The characteristic fluorescence of the quantum dot was observed to localize primarily in the liver, lung and spleen with little or no accumulation in brain, heart or kidney. Consistent with the *in vivo* analysis, a polyarginine CPP associated non-covalently with quantum dots was shown to deliver its cargo to a human broncho-alveolar carcinoma-derived cell line *in vitro* [81]. The blood-brain barrier is a major hurdle for labeling with QDs. To overcome this barrier, TAT-QD conjugates were given intra-arterially to rats with high uptake and localization of fluorescence to the brain within minutes, a feat that could not be accomplished by QDs alone [82].

Additional imaging applications of CPPs are also being developed. Treatment of T cells with a conjugate of TAT CPP and gadolinium resulted in intracellular T1 relaxation enhancement, and in preliminary *in vivo*, T1-weighted MRI experiments, significantly enhanced liver, kidney, and mesenteric signals [83]. The potential of conjugating TAT with technetium-99c, a radio-isotope in common clinical use, has also been investigated [84,85]. Such conjugates have also been explored for imaging of prostate and breast cancer cell lines *in vitro* and have shown good internalization and inhibition of cell proliferation [86,87]. However, these studies utilize a non-specific tracer, technetium-99m, with a non-tissue specific CPP, TAT, that is unlikely to be clinically useful as it would have ubiquitous uptake in whole organisms leading to a high incidence of off-target effects. However, conjugation of tissue specific CPPs to nuclear radioisotopes, such as technetium or thallium, to enhance organ specific uptake with minimization of background signal to give better image resolution with a lower radioisotope dose remains to be explored.

## 7. Hurdles to Clinical Application

It has now been over 25 years since the initial description of proteins being able to transverse cell membranes and the localization of this activity to smaller 6–30 amino acid residues known as cell penetrating peptides (CPPs) or protein transduction domains (PTDs). They have generated significant interest as to their mechanism of transduction, their diagnostic and therapeutic potential for delivering drugs, DNA, siRNA, along with various imaging agents including, but not limited to, radio-isotopes and quantum dots. Yet there are no published human studies utilizing any of the CPPs either as diagnostic or therapeutic agents, although two phase I trials of a CPP (p28) are listed at clinicaltrials.gov as ongoing for treatment of solid tumors expressing p53 and for CNS malignancies. In addition, there are several unpublished industry-sponsored Phase II clinical trials that utilize CPP for delivery (AZX100, Capstone Therapeutics, for keloid scarring; RT001, ReVance Therapeutics, for wrinkling skin; KAI-9803, KAI Pharmaceuticals, for myocardial infarction; XG-102, Auris Medical, for hearing loss).

There clearly are a few hurdles to be overcome before these CPP based therapeutics can be translated into clinical utility, which will be briefly highlighted here. As peptides, CPPs are fairly expensive to synthesize and cost would be an issue, especially when it comes to upscaling for larger animal and eventually human studies. Secondly, these peptides do not have any oral bioavailability and to date have been delivered clinically either through topical, or intravenous applications. The third issue relates to the non-specific uptake of the cationic and hydrophobic CPPs. These earlier CPPs are generally potent and efficient transducers, but also highly non-specific, which makes non-target side effects much more likely and reduces the ratio of therapeutic to side effects. This problem can be addressed by using tissue-specific CPPs that could also, at least theoretically, reduce the dose necessary and make the side effect profile more acceptable due to less non-specific, non-target tissue uptake. Immunogenicity of these peptides, especially following chronic administration, needs to be carefully studied prior to clinical application as was observed following chronic treatment of the dog model of DMD with the Antp-NBD peptide. Given their small size, it is unlikely that the CPPs themselves would elicit an immunogenic response, but their cargo can vary in size and depending on the fusion product being delivered, adverse immune responses might occur following chronic treatment. Lastly, as with any new drug or therapeutic in development, kidney and liver toxicity, as these are usually the two major routes of elimination, needs to be carefully assessed and weighed against the therapeutic benefit.

## 8. Summary

Cell penetrating peptides (CPP), also known as protein transduction domains (PTD), either tissue specific or non-specific, are small peptides able to carry proteins, peptides, nucleic acid, and particles, including viral particles, across the cellular membranes into cells. These peptides can be used for therapeutic applications, such as for improving delivery of proteins, nucleic acid and drugs into cells follow local or systemic delivery. In mouse models of disease, including cancer and inflammatory disease, the effectiveness of CPPs as a delivery method for therapeutic proteins and peptides has been demonstrated. Also, based on mouse studies, CPPs appear useful for more efficient delivery of genomic material including DNA and siRNA. Similarly, CPPs can increase viral infection for gene therapy applications and can be used to facilitate uptake of plasmid DNA. In addition to therapeutic applications, CPPs can be used for delivery of fluorescent or radioactive agents for imaging applications. Taken together, the preclinical studies using CPPs for delivery of a variety of agents to cells *in vivo* suggest that CPPs have exciting clinical potential.
